# Combining plasma Aβ and p-tau217 improves detection of brain amyloid in non-demented elderly

**DOI:** 10.1186/s13195-024-01469-w

**Published:** 2024-05-23

**Authors:** Yoshiki Niimi, Shorena Janelidze, Kenichiro Sato, Naoki Tomita, Tadashi Tsukamoto, Takashi Kato, Kenji Yoshiyama, Hisatomo Kowa, Atsushi Iwata, Ryoko Ihara, Kazushi Suzuki, Kensaku Kasuga, Takeshi Ikeuchi, Kenji Ishii, Kengo Ito, Akinori Nakamura, Michio Senda, Theresa A. Day, Samantha C. Burnham, Leonardo Iaccarino, Michael J. Pontecorvo, Oskar Hansson, Takeshi Iwatsubo

**Affiliations:** 1 Unit for Early and Exploratory Clinical Development, The University of Tokyo Hospital, 7-3-1 Hongo, Bunkyo-Ku, Tokyo, 113-0033 Japan; 2 Clinical Memory Research Unit, Department of Clinical Sciences Malmö, Lund University, Malmö, Sweden; 3 Department of Neuropathology, Graduate School of Medicine, The University of Tokyo, Tokyo, Japan; 4 Department of Aging Research and Geriatric Medicine, Institute of Development, Aging and Cancer, Tohoku University, Sendai, Japan; 5 Department of Geriatric Medicine and Neuroimaging, Tohoku University Hospital, Sendai, Japan; 6 Department of Neurology, National Center of Neurology and Psychiatry, Tokyo, Japan; 7 Department of Clinical and Experimental Neuroimaging, National Center for Geriatrics and Gerontology, Aichi, Japan; 8 Department of Psychiatry, Osaka University Graduate School of Medicine, Osaka, Japan; 9 Graduate School of Health Sciences, Kobe University, Hyogo, Japan; 10 Department of Neurology, Tokyo Metropolitan Institute for Geriatrics and Gerontology, Tokyo, Japan; 11 Division of Neurology, Internal Medicine, National Defense Medical College, Saitama, Japan; 12 Department of Molecular Genetics, Brain Research Institute, Niigata University, Niigata, Japan; 13 Integrated Research Initiative for Living Well With Dementia, Tokyo Metropolitan Institute for Geriatric and Gerontology, Tokyo, Japan; 14 Department of Molecular Imaging Research, Kobe City Medical Center General Hospital, Hyogo, Japan; 15 Eli Lilly and Company, Indianapolis, USA; 16 Memory Clinic, Skåne University Hospital, Lund, Sweden

**Keywords:** Blood-based biomarker, p-tau217, Amyloid-β, Amyloid positron emission tomography

## Abstract

**Background:**

Maximizing the efficiency to screen amyloid-positive individuals in asymptomatic and non-demented aged population using blood-based biomarkers is essential for future success of clinical trials in the early stage of Alzheimer’s disease (AD). In this study, we elucidate the utility of combination of plasma amyloid-β (Aβ)-related biomarkers and tau phosphorylated at threonine 217 (p-tau217) to predict abnormal Aβ-positron emission tomography (PET) in the preclinical and prodromal AD.

**Methods:**

We designed the cross-sectional study including two ethnically distinct cohorts, the Japanese trial-ready cohort for preclinica and prodromal AD (J-TRC) and the Swedish BioFINDER study. J-TRC included 474 non-demented individuals (CDR 0: 331, CDR 0.5: 143). Participants underwent plasma Aβ and p-tau217 assessments, and Aβ-PET imaging. Findings in J-TRC were replicated in the BioFINDER cohort including 177 participants (cognitively unimpaired: 114, mild cognitive impairment: 63). In both cohorts, plasma Aβ(1-42) (Aβ42) and Aβ(1-40) (Aβ40) were measured using immunoprecipitation-MALDI TOF mass spectrometry (Shimadzu), and p-tau217 was measured with an immunoassay on the Meso Scale Discovery platform (Eli Lilly).

**Results:**

Aβ-PET was abnormal in 81 participants from J-TRC and 71 participants from BioFINDER. Plasma Aβ42/Aβ40 ratio and p-tau217 individually showed moderate to high accuracies when detecting abnormal Aβ-PET scans, which were improved by combining plasma biomarkers and by including age, sex and *APOE* genotype in the models. In J-TRC, the highest AUCs were observed for the models combining p-tau217/Aβ42 ratio, *APOE*, age, sex in the whole cohort (AUC = 0.936), combining p-tau217, Aβ42/Aβ40 ratio, *APOE*, age, sex in the CDR 0 group (AUC = 0.948), and combining p-tau217/Aβ42 ratio, *APOE*, age, sex in the CDR 0.5 group (AUC = 0.955), respectively. Each subgroup results were replicated in BioFINDER, where the highest AUCs were seen for models combining p-tau217, Aβ42/40 ratio, *APOE*, age, sex in cognitively unimpaired (AUC = 0.938), and p-tau217/Aβ42 ratio, *APOE*, age, sex in mild cognitive impairment (AUC = 0.914).

**Conclusions:**

Combination of plasma Aβ-related biomarkers and p-tau217 exhibits high performance when predicting Aβ-PET positivity. Adding basic clinical information (i.e., age, sex, *APOE* ε genotype) improved the prediction in preclinical AD, but not in prodromal AD. Combination of Aβ-related biomarkers and p-tau217 could be highly useful for pre-screening of participants in clinical trials of preclinical and prodromal AD.

**Supplementary Information:**

The online version contains supplementary material available at 10.1186/s13195-024-01469-w.

## Background

Alzheimer’s disease (AD) is the most common neurodegenerative disorder and the leading cause of dementia worldwide, threatening aging societies with a vastly increasing number of patients with dementia, and its economic and social burden. Two disease-modifying therapies (DMTs) targeting amyloid-β (Aβ) pathology, aducanumab and lecanemab, have recently been approved by the FDA for use in the early symptomatic stage of AD [1–4]. Another anti-Aβ drug, donanemab, has met the primary and secondary cognitive endpoints in its phase 3 clinical trial [5]. While the slowing of cognitive decline in response to these therapies was modest, results from the donanemab and lecanemab trials [6, 7] suggest that Aβ-targeting DMTs may be more effective in the earliest stages of AD[3, 4, 6, 7]. This will most likely lead to a shift in the target population of future clinical trials of DMTs to preclinical and prodromal AD. However, recruitment of participants in the earliest stages of AD is challenging due to the low prevalence of preclinical AD in cognitively normal individuals and those with subjective cognitive decline [8] and the invasiveness and high cost of the current gold standard markers, i.e., amyloid positron emission tomography (PET) scans and cerebrospinal fluid (CSF) biomarkers. The use of emerging blood-based biomarkers in the screening of potential trial participants has been highlighted as an efficient approach to overcome these limitations [9, 10]. Although promising results on plasma biomarkers, e.g., Aβ(1–42) (Aβ42) reductions [11–13] or increases in phosphorylated tau [14] have recently been reported, the accuracy of the combination of plasma biomarkers in predicting amyloid status in individuals at the preclinical or prodromal stages of AD has not been fully investigated. In addition, the effect of ethnic differences on the predictive power of plasma biomarkers has not been well characterized [15, 16]. In this study, we demonstrated the very high performance of the combination of plasma Aβ and p-tau217 to detect brain Aβ-PET positivity in people with early-stage AD in the Japanese J-TRC cohort, which was replicated in the second Caucasian BioFINDER cohort.

## Methods

### Subjects

Participants were recruited from the J-TRC in-person cohort (J-TRC onsite study), which consists of web-based registry participants (J-TRC webstudy), existing local cohort participants (ORANGE registry)[17], and outpatients from J-TRC organizing institutions (the University of Tokyo Hospital, National Center for Geriatrics and Gerontology, National Center of Neurology and Psychiatry, Tohoku University Hospital, Tokyo Metropolitan Institute for Geriatrics and Gerontology, Osaka University Hospital, Kobe University Hospital). Webstudy participants were invited to a face to face study according to the previously reported algorithm [18]. Individuals with a diagnosis of dementia at enrollment were excluded. Participants were assessed for cognitive and clinical impairment, including the Cognitive Function Instrument (CFI) [19], the Preclinical Alzheimer’s Cognitive Composite (PACC) [20], and the Clinical Dementia Rating (CDR) scales. PACC includes MMSE (Mini-Mental State Examination), WMS (Wechsler Memory Scale) Delayed Recall, WAIS-R (Wechsler Adult Intelligence Scale-Revised) Digit Symbol, and FCSRT (Free and Cued Selective Reminding Test). Participants also underwent blood biomarker testing for Aβ(1–40) (Aβ40) and Aβ(1–42) (Aβ42) (Shimadzu), plasma p-tau217 (Eli Lilly), *APOE* genotyping, and amyloid PET with either [^18^F]-florbetapir (FBP) or [^18^F]-flutemetamol (FMM). Participants diagnosed with preclinical or prodromal AD are followed annually until they are referred to appropriate clinical trials. As of December 2023, ~ 14,000 subjects have consented to participate in the webstudy, and 630 have been invited for in-person assessment. In this study, we evaluated 474 subjects enrolled in the J-TRC onsite study from July 2020 to November 2022. From the BioFINDER cohort [21], we examined 177 participants, all of whom underwent evaluation of Aβ40 and Aβ42 (Shimadzu) and p-tau217 (Eli Lilly) in plasma and Aβ PET imaging with FMM.

### Sample collection and plasma biomarker measurements

On the first day of the J-TRC in-person study, 14 mL of blood was collected from each participant and was placed in two 7 mL vacuum tubes containing 10.5 mg of EDTA.2Na and centrifuged (2000 × g, 5 min, 4 ℃) to obtain plasma samples. Aliquots of 3 ml plasma were immediately frozen at -80 °C and transferred to and stored at the Brain Research Institute, Niigata University. Plasma levels of Aβ42 and Aβ40 were measured by Shimadzu Techno-Research Inc (Kyoto, Japan) using an Immunoprecipitation-Mass Spectrometry (IP-MS)-based method as previously described [11, 22]. Analysis of plasma p-tau217 was performed using the Meso Scale Discovery (MSD) platform at Eli Lilly and Company [21, 23]. In the BioFINDER, plasma levels of Aβ42, Aβ40 and p-tau217 were measured using the same assays as in the J-TRC at Shimadzu Techno-Research (Aβ42 and Aβ40) and Lund University (p-tau217). Details of plasma sampling and biomarker analysis in the BioFINDER are described in previous reports [22, 24].

### Aβ PET imaging

PET scans using 370 ± 74 MBq of FBP or 185 ± 37 MBq of FMM were performed at baseline in all the J-TRC onsite study participants. Acquisition times were 20 min for FBP and 30 min for FMM, starting 50 min (FBP) or 90 min (FMM) after injection of each tracer, followed by image reconstruction using the parameters determined for each PET camera [25]. Aβ PET scan results were interpreted visually by two independent nuclear medicine specialists qualified to read amyloid PET scans in accordance with Japanese guidelines and manufacturers’ instructions, and then by a third rater (adjudicator) if the two raters disagreed. We calculated the centiloid scale using the CapAIBL software package for reference only [26]. In the BioFINDER cohort, scans were acquired 90–110 min after injection of ~ 185 MBq FMM and global standard uptake value ratio (SUVR) values were calculated using the entire cerebellum as the reference region. Aβ PET status was determined by applying a Gaussian mixture model-based threshold of 1.138 to neocortical SUVR values determined in a sample of all BioFINDER 1 participants (N = 445) who underwent FMM PET.

### Statistics

R (version 4.1.0), an open-source software environment, was used for statistical analyses. The chi-square test was used to compare sex, CDR, and the presence of *APOE* ε4 allele status between groups. The Shapiro–Wilk test was used to test the distribution of numerical variables. The Wilcoxon rank-sum test was used to compare variables with non-normal distributions, and Student’s t-test was used to compare variables with normal and equal distributions between groups. Receiver operator characteristic (ROC) analysis was used to assess the ability of each biomarker to predict Aβ-PET positivity. Cutoff values were determined using a Youden index. The DeLong test was used to compare area under the curve (AUC) metrics from two ROC evaluations. To investigate the improvement in accuracy for predicting Aβ-PET positivity by combining plasma biomarkers with age, sex, and *APOE*, we applied a logistic regression model. The Akaike Information Criterion (AIC) was calculated to assess model fit. Statistical significance was set at p < 0.05. The Benjamini–Hochberg correction was applied for multiple comparisons.

## Results

### The J-TRC cohort

#### Participants

The characteristics of the participants from the J-TRC cohort, including the comparison between the Aβ-PET positive and negative groups, are shown in Table 1. Of the 474 participants in the J-TRC cohort, the visual interpretation by the two raters agreed in 94% of the cases, and 81 were classified as Aβ-PET positive, with global CDR scores of 0 in 331 participants and 0.5 in 143 participants. The rate of *APOE* ε4 allele carrier (one or two alleles) in the entire cohort was 20.3%. Compared with the Aβ negative group, the Aβ-PET positive group had a significantly higher mean age, prevalence of *APOE* ε4 allele positivity, and proportion of participants with a CDR 0.5, while no significant differences were found for sex or education. The Aβ-PET positive group also had worse average cognitive scores as assessed by MMSE, WMS Delayed Recall, WAIS-R Digit Symbol, FCSRT, and CFI. As expected, the Aβ-PET positive group had a significantly lower plasma Aβ42/Aβ40 (Aβ42/40) ratio and higher p-tau217 levels (Table 1a). The CDR 0.5 group had lower educational attainment and more severe cognitive impairment, whereas no differences were found in the rate of *APOE* ε4 allele carrier compared with the CDR 0 group (Table 1b).

**Table 1:** Demographics of participant

**a) Participants’ demographics by brain amyloid PET result**							
		**J-TRC**			**BioFINDER**		
		**Aβ-PET negative**	**Aβ-PET positive**	**p-value**	**Aβ-PET negative**	**Aβ-PET positive**	**p-value**
N, (%)		393(83)	81(17)		108(61)	69(39)	
PET tracer; FBP/FMM, (%)		180(46)/213(54)	22(27)/59(73)		0/108	0/69	
Age (mean ± SD, years)		71.2 ± 6.5	73.5 ± 6.3	**0.0015**	71.5 ± 5.9	73.5 ± 4.9	**0.023**
Male/Female, (%)		224(57)/169(43)	44(54)/37(46)	N.S	49(45)/59(55)	34(49)/35(51)	N.S
Education (mean ± SD, years)		14.4 ± 2.4	14.1 ± 2.8	N.S	11.9 ± 3.5	11.0 ± 3.0	N.S
*APOE* ε4 +/- , (%)		61(16)/332(84)	35(43)/46(57)	** < 0.001**	22(21)/84(79)	50(72)/19(28)	**< 0.001**
CDR 0/CDR 0.5 (J-TRC), (%) CU/MCI (BioFINDER), (%)		289(74)/104(26)	42(52)/39(48)	** < 0.001**	83(77)/25(23)	31(45)/38(55)	**< 0.001**
MMSE (mean ± SD)		28.2 ± 1.9	27.4 ± 2.6	**0.029**	28.4 ± 1.6	27.6 ± 1.7	**0.001**
WMS Logical Memory IIa; Delayed Recall (mean ± SD)		8.6 ± 4.2	6.2 ± 4.9	** < 0.001**	N.A	N.A	
WAIS-R; Digit Symbol Substitution Test (mean ± SD)		49.6 ± 12.2	43.9 ± 11.2	** < 0.001**	N.A	N.A	
FCSRT (mean ± SD)		46.7 ± 3.2	43.7 ± 6.9	** < 0.001**	N.A	N.A	
CFI (mean ± SD)	Self	3.4 ± 2.4	4.7 ± 3.0	** < 0.001**	N.A	N.A	
	Study partner	1.6 ± 1.7	2.7 ± 2.6	** < 0.001**	N.A	N.A	
Plasma Aβ42/40 (mean ± SD)		0.043 ± 0.009	0.034 ± 0.006	** < 0.001**	0.053 ± 0.006	0.046 ± 0.004	** < 0.001**
Plasma p-tau217 (mean ± SD, pg/ml)		0.16 ± 0.08	0.33 ± 0.17	** < 0.001**	0.17 ± 0.06	0.32 ± 0.14	** < 0.001**
PET Centiloid Scale (mean ± SD)		-0.19 ± 11.46	52.02 ± 31.14	** < 0.001**	N.A	N.A	
**b) Participants’ demographics by cognitive assessment**							
		**J-TRC**			**BioFINDER**		
		**CDR 0**	**CDR 0.5**	**p-value**	**CU**	**MCI**	**p-value**
N, (%)		331(70)	143(30)		114(64)	63(36)	
Age (mean ± SD, years)		70.7 ± 6.4	73.8 ± 6.3	** < 0.001**	72.9 ± 5.4	71.1 ± 5.8	**0.036**
Male/Female, (%)		191(58)/140(42)	77(54)/66(46)	N.S	43(38)/71(62)	40(63)/23(37)	**0.001**
Education (mean ± SD, years)		14.5 ± 2.5	13.8 ± 2.5	**0.0075**	12.1 ± 3.2	10.6 ± 3.4	**0.004**
*APOE* ε4 +/- , (%)		59(18)/272(82)	37(26)/106(74)	N.S	42(38)/70(64)	30(48)/33(52)	N.S
Aβ-PET -/+ , (%)		289(87)/42(13)	104(73)/39(27)	** < 0.001**	83(73)/31(27)	25(40)/38(60)	** < 0.001**
MMSE (mean ± SD)		28.5 ± 1.7	27 ± 2.4	** < 0.001**	28.7 ± 1.3	27.0 ± 1.7	** < 0.001**
WMS Logical Memory IIa; Delayed Recall (mean ± SD)		9.2 ± 4.1	5.8 ± 4.3	** < 0.001**	N.A	N.A	
WAIS-R; Digit Symbol Substitution Test (mean ± SD)		50.8 ± 11.8	43.7 ± 11.8	** < 0.001**	N.A	N.A	
FCSRT (mean ± SD)		47.1 ± 1.5	44.1 ± 7.0	** < 0.001**	N.A	N.A	
CFI (mean ± SD)	Self	3.1 ± 2.2	4.8 ± 2.9	** < 0.001**	N.A	N.A	
	Study partner	1.3 ± 1.4	2.8 ± 2.4	** < 0.001**	N.A	N.A	
Plasma Aβ42/40 (mean ± SD)		0.043 ± 0.009	0.041 ± 0.012	**0.0023**	0.051 ± 0.006	0.048 ± 0.005	** < 0.001**
Plasma p-tau217 (mean ± SD, pg/ml)		0.17 ± 0.08	0.24 ± 0.18	** < 0.001**	0.20 ± 0.09	0.28 ± 0.15	** < 0.001**

*p*-value were adjusted by Benjamini–Hochberg method

*FBP* [^18^F]-florbetapir, *FMM* [^18^F]-flutemetamol, *CDR* Clinical dementia rating (global score), *CU* Cognitively unimpaired, *MCI* Mild cognitive impairment, *FCSRT* Free and cued selective reminding test, *WMS* Wechsler memory scale, *WAIS-R* Wechsler adult intelligence scale-revised, *MMSE* Mini-mental state examination, *CFI* Cognitive function instrument. *N.A*. not available

### Prediction of Aβ PET status using plasma biomarkers

**Fig. 1:**
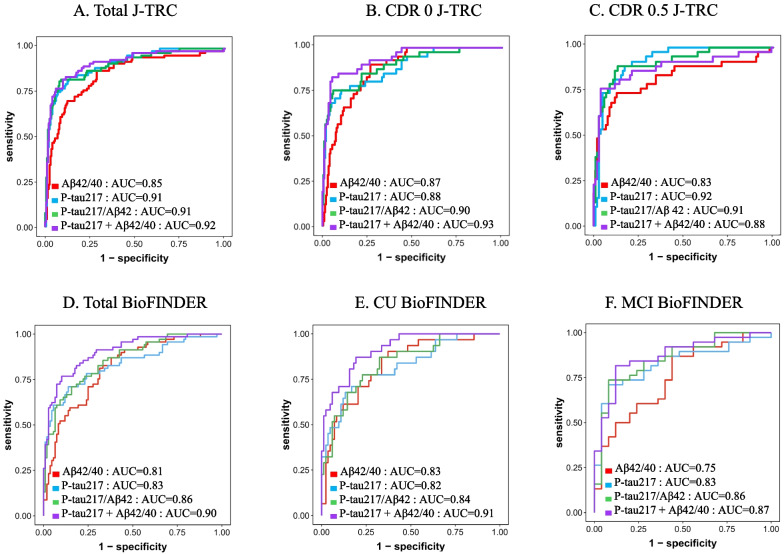
ROC curve analysis for the detection of amyloid PET positivity. ROC curve analysis in the total participants from the J-TRC cohort (n = 474) (**A**), in the CDR 0 participants from the J-TRC cohort (*n* = 331) (**B**), and in the CDR 0.5 participants from the J-TRC cohort (n = 143) (**C**), in the total participants from the BioFINDER cohort (*n* = 177) (**D**), in the CU participants from the BioFINDER cohort (*n* = 114) (**E**) and in the MCI participants from the BioFINDER cohort (*n* = 63) (**F**). CDR: clinical dementia rating (global score), CU: cognitively unimpaired, MCI: mild cognitive impairment

We first evaluated the performance of plasma biomarkers, e.g., Aβ42/40 ratio, p-tau217, p-tau217/Aβ42 ratio, and the combination of plasma p-tau217 and Aβ42/40 ratio, to identify individuals with abnormal Aβ-PET scans (Fig. 1). In the entire J-TRC cohort, all tested plasma biomarker models showed moderate to high accuracy (AUCs ranging from 0.856 (95%CI: 0.808–0.904) for the Aβ42/40 ratio to 0.920 (95%CI: 0.884–0.957) for the combination of plasma p-tau217 and Aβ42/40 ratio). The AUC values including the blood biomarkers showed higher AUCs compared to those combining the cognitive screening test with clinical information, e.g., MMSE with age, sex, and *APOE* status (AUC 0.721 (95%CI: 0.660–0.782)). The combination of plasma p-tau217 and Aβ42/40 ratio had a significantly higher AUC than the Aβ42/40 ratio alone (p < 0.001, Table 2). We next examined the discriminative accuracy of the plasma biomarkers for Aβ PET status separately in the CDR 0 and CDR 0.5 groups. In the CDR 0 group, all tested plasma biomarker models showed moderate to high accuracy AUCs ranging from 0.876 (95%CI: 0.831–0.922) for Aβ42/40 ratio to 0.938 (95%CI: 0.902–0.975) for the combination of plasma p-tau217 and Aβ42/40 ratio. The combination of plasma p-tau217 and Aβ42/40 ratio showed a significantly higher AUC compared to the other AUCs (*p* = 0.013 for Aβ42/40 ratio, *p* = 0.012 for p-tau217, *p* = 0.049 for p-tau217/Aβ42 ratio, Table 2). In the CDR 0.5 group, plasma biomarkers also showed moderate to high accuracy (AUC ranging from 0.830 (95%CI: 0.740–0.920) for Aβ42/40 ratio to 0.925 (95%CI: 0.881–0.969) for p-tau217). However, in contrast to the results within the entire cohort and the CDR 0 group, the AUCs of the combination of plasma p-tau217 and Aβ42/40 ratio in the CDR 0.5 group were not significantly different from other AUCs (Table 2).

**Table 2:** Results of the DeLong test comparing AUC of combinations of biomarkers for predicting amyloid PET positivity in the J-TRC cohort

		p-tau217	p-tau217/Aβ42	p-tau217 + Aβ42/40	Aβ42/40 + CI	p-tau217 + CI	p-tau217/Aβ42 + CI	p-tau217 + Aβ42/40 + CI
ALL	Aβ42/40	N.S	N.S	** < 0.001**	N.S	N.S	**0.0092**	** < 0.001**
	p-tau217		N.S	N.S	N.S	N.S	N.S	N.S
	p-tau217/Aβ42			N.S	N.S	N.S	N.S	N.S
	p-tau217 + Aβ42/40				**0.0010**	N.S	N.S	N.S
	Aβ42/40 + CI					N.S	**0.014**	** < 0.001**
	p-tau217 + CI						N.S	N.S
	p-tau217/Aβ42 + CI							N.S
CDR 0	Aβ42/40	N.S	N.S	**0.013**	N.S	N.S	N.S	**0.016**
	p-tau217		N.S	**0.012**	N.S	N.S	**0.034**	**0.012**
	p-tau217/Aβ42			**0.049**	N.S	N.S	N.S	N.S
	p-tau217 + Aβ42/40				**0.032**	N.S	N.S	N.S
	Aβ42/40 + CI					N.S	N.S	**0.093**
	p-tau217 + CI						N.S	**0.035**
	p-tau217/Aβ42 + CI							N.S
CDR 0.5	Aβ42/40	N.S	N.S	N.S	N.S	N.S	N.S	N.S
	p-tau217		N.S	N.S	N.S	N.S	N.S	N.S
	p-tau217/Aβ42			N.S	N.S	N.S	N.S	N.S
	p-tau217 + Aβ42/40				N.S	N.S	N.S	N.S
	Aβ42/40 + CI					N.S	N.S	N.S
	p-tau217 + CI						N.S	N.S
	p-tau217/Aβ42 + CI							N.S

*CI* Clinical information including age, sex, and *APOE*, *CDR* Clinical dementia rating (global score)

*p*-values were adjusted by Benjamini–Hochberg method. N.S.: not significant

### Prediction of Aβ PET status using plasma biomarkers and age, sex, and *APOE*

**Fig. 2:**
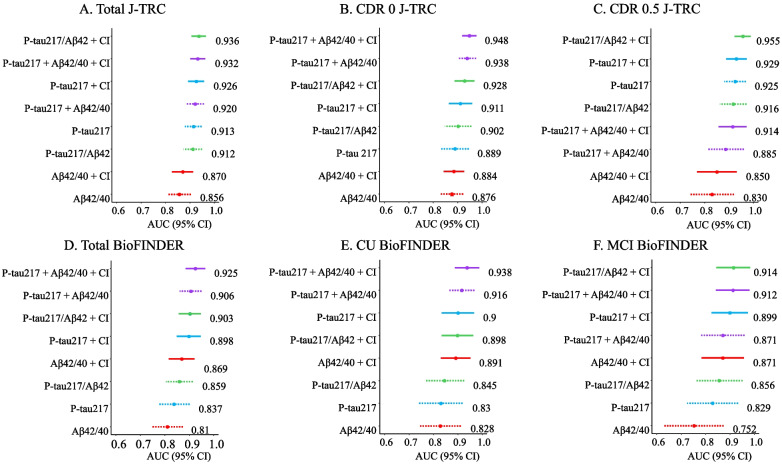
Ranking of different biomarker combination models sorted by the AUC. AUC (95% CI) ranking in the total participants from the J-TRC cohort (*n* = 474) (**A**), in the CDR 0 participants from the J-TRC cohort (*n* = 331) (**B**), in the CDR 0.5 participants from the J-TRC cohort (*n* = 143) (**C**), in the total participants from the BioFINDER cohort (*n* = 177) (**D**), in the CU participants from the BioFINDER cohort (*n* = 114) (**E**), in the MCI participants form the BioFINDER cohort (*n* = 63) (**F**). Dotted lines represent the results for biomarkers, while solid lines represent the results for biomarkers with clinical information. AUC values are shown in the right side of each line. CI: clinical information including age, sex, and *APOE*, CDR: clinical dementia rating (global score), CU: cognitively unimpaired, MCI: mild cognitive impairment

We next tested whether combining age, sex, and *APOE* genotype with the plasma biomarker improved their ability to detect Aβ-PET positivity, with results shown in Table 2. The addition of age, sex, and *APOE* genotype nominally increased the AUCs for all models tested in the J-TRC cohort, regardless of the CDR values (Table 3). The DeLong test among all models, e.g., biomarkers alone and a model with clinical information (CI) including age, sex, and *APOE*, showed that in the CDR 0 group, the combination of p-tau217 and Aβ42/40 ratio with CI was superior to Aβ42/40 or p-tau217 alone and with CI. In contrast, there was no significant difference in AUCs in the CDR 0.5 group (Table 2). The ranking of the different models sorted by the AUC is shown in Fig. 2. The results obtained from the J-TRC cohort showed that the combination of p-tau217 with Aβ42/40 ratio with age, sex, and *APOE* had the highest AUC for detecting Aβ-PET positivity in the CDR 0 participants, whereas a model combining p-tau217/Aβ42 ratio with age, sex, and *APOE* was superior to other models in the CDR 0.5 population. Furthermore, in the CDR 0 group, the AIC value of the combination of p-tau217 with Aβ42/40 ratio, or the combination of p-tau217 with Aβ42/40 ratio and CI was lower compared to the models including other plasma biomarkers (Table 3). At the same time, the AIC of p-tau217/Aβ42 ratio with CI was the smallest in the CDR 0.5 group. The results of AIC support that these combinations fit better than any other biomarker modeling in each group.

**Table 3:** AUC, sensitivity, specificity, PPV, NPV, AIC by individual modeling in the J-TRC cohort

		AUC (95% CI)	sensitivity	specificity	PPV	NPV	AIC
a)	Total (N = 474)						
	Aβ42/40	0.856 (0.808–0.904)	0.876	0.712	0.385	0.965	322.07
	Aβ42/40 + Age + Sex + *APOE*	0.870 (0.827–0.913)	0.851	0.743	0.405	0.960	311.73
	p-tau217	0.913 (0.879–0.948)	0.839	0.865	0.561	0.963	299.84
	p-tau217 + Age + Sex + *APOE*	0.926 (0.893–0.959)	0.901	0.862	0.574	0.976	276.36
	p-tau217/Aβ42	0.912 (0.874–0.950)	0.814	0.923	0.687	0.960	247.6
	p-tau217/Aβ42 + Age + Sex + *APOE*	0.936 (0.906–0.965)	0.827	0.921	0.683	0.962	218.26
	p-tau217 + Aβ42/40	0.920 (0.884–0.957)	0.827	0.900	0.632	0.961	250.51
	p-tau217 + Aβ42/40 + Age + Sex + *APOE*	0.932 (0.901–0.963)	0.975	0.737	0.434	0.993	237.99
b)	CDR 0 group (N = 331)						
	Aβ42/40	0.876 (0.831–0.922)	0.904	0.733	0.330	0.981	185.67
	Aβ42/40 + Age + Sex + *APOE*	0.884 (0.842–0.927)	0.976	0.657	0.292	0.994	183.43
	p-tau217	0.889 (0.832–0.945)	0.761	0.896	0.516	0.962	153.01
	p-tau217 + Age + Sex + *APOE*	0.911 (0.863–0.96)	0.857	0.837	0.433	0.975	147.68
	p-tau217/Aβ42	0.902 (0.847–0.956)	0.761	0.941	0.653	0.964	146.24
	p-tau217/Aβ42 + Age + Sex + *APOE*	0.928 (0.888–0.968)	0.809	0.896	0.531	0.970	140.11
	p-tau217 + Aβ42/40	0.938 (0.902–0.975)	0.833	0.944	0.686	0.975	129.83
	p-tau217 + Aβ42/40 + Age + Sex + *APOE*	0.948 (0.919–0.977)	0.952	0.813	0.425	0.991	130.35
c)	CDR 0.5 group (N = 143)						
	Aβ42/40	0.830 (0.74–0.920)	0.743	0.875	0.690	0.900	133.86
	Aβ42/40 + Age + Sex + *APOE*	0.850 (0.768–0.931)	0.820	0.826	0.64	0.924	132.74
	p-tau217	0.925 (0.881–0.969)	0.897	0.826	0.660	0.955	129.55
	p-tau217 + Age + Sex + *APOE*	0.929 (0.887–0.972)	0.846	0.903	0.767	0.94	117.24
	p-tau217/Aβ42	0.916 (0.862–0.970)	0.897	0.865	0.714	0.957	100.44
	p-tau217/Aβ42 + Age + Sex + *APOE*	0.955 (0.922–0.989)	0.923	0.913	0.8	0.999	80.93
	p-tau217 + Aβ42/40	0.885 (0.810–0.960)	0.769	0.961	0.882	0.917	109.99
	p-tau217 + Aβ42/40 + Age + Sex + *APOE*	0.914 (0.856–0.971)	0.846	0.884	0.733	0.938	103.13

*AUC* Area under curve, *BM* Biomarker, *PPV* Positive predictive value, *NPV* Negative predictive value, *AIC* Akaike’s information criterion

Sensitivity, specificity, ppv, and npv were determined by using the Youden index

### Validation in the BioFINDER cohort

We then sought to validate the results from the J-TRC cohort using data from the Swedish BioFINDER study (Tables 1, 4 and 5, Fig. 2D, E, and F). The relative proportions of the cognitively unimpaired (equal to CDR 0) and MCI (Mild cognitive impairment) (equal to CDR 0.5) were not different compared to J-TRC. The proportions of the Aβ-PET positive participants and *APOE* ε4-positive participants were higher in the BioFINDER cohort. ROC analysis showed that the four variables had moderate to high accuracy, similar to the J-TRC cohort (Fig. 1). In the cognitively unimpaired group, the combination of p-tau217 and Aβ42/40 ratio showed a higher AUC compared to p-tau217 and p-tau217/Aβ42 ratio (Table 4). In the MCI group, there were no significant differences in AUCs between the tested biomarker models. These results were similar to those in the J-TRC cohort (Tables 2 and 3). The results of the ROC analysis (Fig. 2D, E, and F) showed that the combination of p-tau217 and Aβ42/40 ratio with CI had the highest AUC in the cognitively unimpaired group, followed by p-tau217 and Aβ42/40 ratio. This order was identical to that observed in the CDR 0 group of the J-TRC cohort. In the MCI group, the p-tau217/Aβ42 ratio with CI exhibited the highest AUC similar to the findings in the J-TRC. In the cognitively unimpaired group, the AIC value of the combination of p-tau217 with Aβ42/40 ratio, or the combination of p-tau217 with Aβ42/40 ratio and CI, was smaller compared to the models including other plasma biomarkers, as in J-TRC (Table 5). Also, the AIC of p-tau217/Aβ42 ratio with CI was the smallest in the MCI group. These results showed that each model fit better compared to other models, similar to the J-TRC cohort. However, some differences were observed in the performance of variables between J-TRC and BioFINDER in the MCI group: p-tau217 was the third best in the J-TRC, whereas it was the seventh best in the BioFINDER cohort (AUC: 0.925 and 0.829, respectively).

**Table 4:** Results of the DeLong test comparing the AUC of combinations of biomarkers for predicting amyloid PET positivity in BioFINDER

		p-tau217	p-tau217/Aβ42	p-tau217 + Aβ42/40	Aβ42/40 + CI	p-tau217 + CI	p-tau217/Aβ42 + CI	p-tau217 + Aβ42/40 + CI
ALL	Aβ42/40	N.S	N.S	**0.003**	**0.025**	**0.040**	**0.024**	**0.002**
	p-tau217		N.S	**0.016**	N.S	**0.019**	**0.022**	**0.003**
	p-tau217/Aβ42			**0.030**	N.S	N.S	**0.036**	**0.010**
	p-tau217 + Aβ42/40				N.S	N.S	N.S	N.S
	Aβ42/40 + CI					N.S	N.S	**0.022**
	p-tau217 + CI						N.S	**0.035**
	p-tau217/Aβ42 + CI							N.S
CU	Aβ42/40	N.S	N.S	N.S	N.S	N.S	N.S	**0.045**
	p-tau217		N.S	**0.045**	N.S	N.S	N.S	**0.033**
	p-tau217/Aβ42			**0.045**	N.S	N.S	N.S	**0.033**
	p-tau217 + Aβ42/40				N.S	N.S	N.S	N.S
	Aβ42/40 + CI					N.S	N.S	N.S
	p-tau217 + CI						N.S	N.S
	p-tau217/Aβ42 + CI							N.S
MCI	Aβ42/40	N.S	N.S	N.S	N.S	N.S	N.S	N.S
	p-tau217		N.S	N.S	N.S	N.S	N.S	N.S
	p-tau217/Aβ42			N.S	N.S	N.S	N.S	N.S
	p-tau217 + Aβ42/40				N.S	N.S	N.S	N.S
	Aβ42/40 + CI					N.S	N.S	N.S
	p-tau217 + CI						N.S	N.S
	p-tau217/Aβ42 + CI							N.S

*CU* Cognitively unimpaired, *MCI* Mild cognitive impairment. p-values were adjusted by the Benjamini–Hochberg method. N.S.: not significant

**Table 5:** AUC, sensitivity, specificity, PPV, NPV, AIC by individual modeling in the BioFINDER cohort

		AUC (95% CI)	sensitivity	specificity	PPV	NPV	AIC
a)	Total (N = 175)^a^						
	Aβ42/40	0.810 (0.746–0.874)	0.826	0.670	0.620	0.855	184.47
	Aβ42/40 + Age + Sex + *APOE*	0.869 (0.816–0.921)	0.797	0.792	0.714	0.857	165.09
	p-tau217	0.837 (0.773–0.902)	0.710	0.868	0.778	0.821	164.45
	p-tau217 + Age + Sex + *APOE*	0.898 (0.849–0.947)	0.826	0.840	0.770	0.881	144.57
	p-tau217/Aβ42	0.859 (0.804–0.915)	0.710	0.849	0.754	0.818	162.24
	p-tau217/Aβ42 + Age + Sex + *APOE*	0.903 (0.857–0.948)	0.841	0.821	0.753	0.888	142.97
	p-tau217 + Aβ42/40	0.906 (0.861–0.950)	0.768	0.896	0.828	0.856	138.40
	p-tau217 + Aβ42/40 + Age + Sex + *APOE*	0.925 (0.886–0.965)	0.870	0.887	0.833	0.913	129.28
b)	CU group (N = 112)						
	Aβ42/40	0.828 (0.744–0.912)	0.871	0.667	0.500	0.931	103.10
	Aβ42/40 + Age + Sex + *APOE*	0.891 (0.830–0.952)	0.871	0.778	0.600	0.940	91.23
	p-tau217	0.830 (0.740–0.919)	0.677	0.877	0.677	0.877	96.34
	p-tau217 + Age + Sex + *APOE*	0.900 (0.833–0.967)	0.839	0.827	0.650	0.931	86.23
	p-tau217/Aβ42	0.845 (0.765–0.926)	0.774	0.778	0.571	0.900	96.73
	p-tau217/Aβ42 + Age + Sex + *APOE*	0.898 (0.834–0.963)	0.839	0.827	0.650	0.931	86.59
	p-tau217 + Aβ42/40	0.916 (0.864–0.968)	0.903	0.765	0.596	0.954	78.36
	p-tau217 + Aβ42/40 + Age + Sex + *APOE*	0.938 (0.888–0.987)	0.839	0.926	0.813	0.938	73.06
c)	MCI group (N = 63)						
	Aβ42/40	0.752 (0.629–0.874)	0.868	0.560	0.750	0.737	75.60
	Aβ42/40 + Age + Sex + *APOE*	0.871 (0.784–0.957)	0.868	0.760	0.846	0.792	67.26
	p-tau217	0.829 (0.725–0.934)	0.711	0.920	0.931	0.676	65.91
	p-tau217 + Age + Sex + *APOE*	0.899 (0.824–0.974)	0.895	0.800	0.872	0.833	59.35
	p-tau217/ Aβ42	0.856 (0.759–0.952)	0.737	0.920	0.933	0.697	64.05
	p-tau217/ Aβ42 + Age + Sex + *APOE*	0.914 (0.844–0.983)	0.895	0.800	0.872	0.833	57.54
	p-tau217 + Aβ42/40	0.871 (0.781–0.960)	0.816	0.880	0.912	0.759	61.84
	p-tau217 + Aβ42/40 + Age + Sex + *APOE*	0.912 (0.843–0.980)	0.921	0.760	0.854	0.864	59.83

^a^Data are from a sample of 175 participants from the BioFINDER cohort (excluding 2 CU participants with missing *APOE* genotype data)

*CU* Cognitively unimpaired, *MCI* Mild cognitive impairment, *AUC* Area under curve, *BM* biomarker, *PPV* positive predictive value, *NPV* Negative predictive value, *AIC* Akaike’s information criterion

Sensitivity, specificity, ppv, and npv were determined by using the Youden index

## Discussion

In this study, we have shown that the plasma Aβ42/40 ratio determined by IP/MS and plasma p-tau217 measured by MSD immunoassay are biomarkers that predict brain Aβ PET positivity in the Japanese population of non-demented individuals, and that a combination of these Aβ42 and p-tau217 markers showed unprecedented high discriminative values with an AUC of ~ 0.93, which was reproduced in the European BioFINDER cohort. Emerging evidence supports the importance of newer blood-based markers in the detection of cerebral Aβ pathology [10, 22]. In a head-to-head comparison study of several plasma Aβ assays, MS-based plasma Aβ biomarkers were reported to be generally superior to immunoassays in detecting abnormal brain amyloid status: IP-MS method developed by Washington University showed the highest AUC of 0.852 for CSF Aβ42/40 status in cognitively unimpaired and MCI subjects, which was improved to 0.882 by the addition of *APOE* genotype [22]. Here we showed that the plasma Aβ42/40 ratio, as determined by the Shimadzu-developed IP-MS assay, predicted brain Aβ PET positivity in the J-TRC cohort, which enrolled non-demented elderly individuals by consecutive recruitment through web-based participation and local cohort or memory clinic, thus reflecting the characteristics of elderly individuals in the general population. Plasma Aβ42/40 measures have been shown to be highly discriminative of CSF Aβ42/40 and Aβ PET status, as well as predictive of cognitive decline and progression from cognitively unimpaired to MCI and from MCI to AD [11, 12, 27]. Changes in the plasma Aβ42/40 ratio precede elevated amyloid levels detected by PET scans, similar to those in CSF [9, 28]. Notably, plasma Aβ42/40 showed moderate accuracy in both the cognitively unimpaired (CDR 0) and the MCI (CDR 0.5) subjects, while the accuracy of the plasma p-tau217 was higher, and in the CDR 0.5 group, p-tau217 showed high accuracy (AUC 0.925). It has been well documented that high levels of plasma p-tau, especially p-tau217, are associated with abnormal Aβ PET and CSF Aβ42/40 in different stages of AD [9, 21, 28, 29]. Elevated plasma p-tau levels have been shown to be highly specific for brain amyloid deposition, allowing the differentiation of AD from non-AD dementia [21, 23, 30–32]. Furthermore, a recent study suggests that plasma p-tau has a strong surrogacy in preclinical and prodromal AD compared to Aβ42/40 ratio or p-tau231 [33]. The superiority of p-tau217 over Aβ42/40 ratio, especially in the prodromal population, is confirmed in BioFINDER. Furthermore, our study suggested an interesting difference in the predictive ability between Aβ42/40 ratio and p-tau217. As shown in Table 3, Aβ42/40 ratio showed relatively higher sensitivity (0.904) and lower specificity (0.733) in the CDR 0 group, while the results were opposite in the CDR 0.5 group (0.743, 0.875, respectively). P-tau217 showed the opposite result (sensitivity 0.761, specificity 0.896 in the CDR 0 group while 0.897 and 0.826, respectively, in the CDR 0.5 group). These results indicate that when blood biomarker is used as a pre-screen for amyloid PET, p-tau217 reduces false positive compared to Aβ42/40 ratio in the CDR 0 group, while Aβ42/40 ratio may be better compared to p-tau217 in the CDR 0.5 group. These results are not replicated in the BioFINDER cohort, where p-tau217 showed higher specificity compared to Aβ42/40 ratio regardless of group. This difference may be due to the difference in disease progression between CDR 0.5 in J-TRC and MCI in BioFINDER, as shown by the plasma p-tau217 level being higher in MCI in BioFINDER (0.28 ± 0.15) compared to CDR 0.5 group in J-TRC (0.24 ± 0.18).

Our results also suggest an intriguing difference in the optimal combination of plasma Aβ42, Aβ42/40, p-tau217 and clinical information in detecting abnormal Aβ PET between the CDR 0 and 0.5 populations. The combination of p-tau217 and Aβ42/40 ratio showed the best performance in the CDR 0 population, while the p-tau217/Aβ42 ratio performed best in the CDR 0.5 group. These results were reproduced in BioFINDER. One interpretation of these findings would be that the plasma Aβ42/40 ratio and p-tau217 gradually change with the progression of brain amyloid accumulation before the threshold of Aβ-PET positivity is reached; the optimal combination of Aβ and p-tau markers may change with disease progression. The benefit of combining plasma Aβ and p-tau biomarkers has not been extensively studied. The superiority of the combination of high performance plasma p-tau217 and Aβ42/40 assays to identify brain Aβ positivity, predict the presence of AD neuropathologic changes [34], Aβ PET centiloid metric [35], and future development of AD dementia has only been reported [36]. Our study demonstrated that the combination of p-tau217 and Aβ42/40 ratio, and these with age, sex, and *APOE* genotype, is a useful tool for predicting Aβ-PET positivity in the cognitively unimpaired or preclinical population. The contribution of each variable to the modeling is shown by a nomogram in Supplementary Figs. 1 and 2.

In many of the previous studies, participants recruited from regional or clinical cohorts included individuals at high-risk for AD with a higher prevalence of the *APOE* ε4 allele (e.g., 45.2% in AHEAD 3–45 study [35], 47.5% in the BioFINDER study [23], 53.9% in the ALFA + cohort [32] and 47.8% in the Wisconsin Registry for Alzheimer’s Prevention cohort [31]), whereas the *APOE* ε4 positivity in the J-TRC cohort was 20.2%, which is close to that of the general population in Japan and Asia [37]. Our study used Aβ-PET positivity as the standard of truth, which was determined by visual interpretation in J-TRC and rated with quantitative measures in BioFINDER. While quantitative methods are popular in Europe, visual assessment is the standard in Japan, as indicated by Japanese guidelines and regulations. As a result, there is variation in amyloid PET interpretation criteria depending on the research protocol. This study examined the reproducibility in two cohorts, each with its own protocol including Aβ-PET rating method. In general, visual and quantitative ratings are known to provide comparable results, allowing for error in borderline cases and taking into account possible differences in case of localized uptake. In this study, the inter-rater variability of the visual reading of the J-TRC was low; the two independent raters agreed in 94% of cases. Also, quantitative measures are not free from variation due to bias, e.g., selection of a software program and a cutoff level. Therefore, the difference in PET rating method between the two cohorts would not substantially affect the conclusions of our study.

Our results may be relevant to the use of plasma biomarkers for prescreening in clinical trials of preclinical AD populations in the real world. Our study also addresses the potential impact of ethnic factors on the usability of plasma biomarkers [15, 38]. Our results showed high performance of the combination of plasma Aβ42, Aβ42/40 ratio, and p-tau217 in non-demented elderly individuals in Asian-Japanese as well as Western populations. Thus, these biomarkers could greatly facilitate the prescreening of participants in global preclinical AD trials that require the enrollment of participants from diverse ethnicities. In addition, these blood-based biomarkers may play an important role in the early detection of individuals at high risk of developing AD and for the early and appropriate diagnosis of cognitive decline or dementia due to AD.

This study has several limitations. Other promising plasma biomarkers such as other phosphorylated tau species (e.g., p-tau231), phosphorylated/non-phosphorylated tau ratio, GFAP, NFL should also be investigated. Our study population of CDR 0.5 in J-TRC and MCI in BioFINDER is relatively small. The rate of *APOE* ε4 allele carriers is not the same in the two cohorts. Thus, the generalizability of our findings, e.g., the difference of optimal combination for cognitively impaired or MCI population in the real-world setting, should be verified in future studies.

## Conclusions

This study demonstrated a high accuracy of the combination of plasma Aβ markers and p-tau217 to detect Aβ-PET positivity in preclinical and prodromal AD in the Japanese trial-ready cohort, which was replicated in the Swedish BioFINDER cohort. These results provide us with optimal indices to identify potential participants and minimize the financial and physical burden of clinical trials of DMTs in the very early stages of AD.

### Supplementary Information

Supplementary Material 1. Supplemental Figure 1 Nomograms for the logistic regression analysis to detect Aβ-PET positivity in the J-TRC cohort. CI: clinical information including age, sex, and *APOE*, CDR: clinical dementia rating (global score). Supplemental Figure 2 Nomograms for the logistic regression analysis to detect Aβ-PET positivity in the BioFINDER cohort. CI: clinical information including age, sex, and *APOE*, CU: cognitively unimpaired, MCI: mild cognitive impairment.

## Data Availability

No datasets were generated or analysed during the current study.

## Abbreviations

Aβ: Amyloid-β
AD: Alzheimer’s disease
AIC: Akaike information criterion
AUC: Area under the curve
CDR: Clinical dementia rating
CFI: Cognitive function instrument
CI: Clinical information
CSF: Cerebrospinal fluid
DMT: Disease-modifying therapy
FBP: [^18^F]-florbetapir
FCSRT: Free and cued selective reminding test
FMM: [^18^F]-flutemetamol
IP-MS: Immunoprecipitation-mass spectrometry
J-TRC: Japanese trial-ready cohort for preclinical and prodromal AD
MCI: Mild cognitive impairment
MMSE: Mini-mental state examination
MSD: Meso scale discovery
PACC: Preclinical Alzheimer’s cognitive composite
PET: Positron emission tomography
ROC: Receiver operator characteristic
SUVR: Standard uptake value ratio
WAIS-R: Wechsler adult intelligence scale-revised
WMS: Wechsler memory scale

## References

[1] Cummings J, Aisen P, Apostolova LG, Atri A, Salloway S, Weiner M (2021). Aducanumab: Appropriate Use Recommendations. J Prev Alzheimers Dis.

[2] Cummings J, Apostolova L, Rabinovici GD, Atri A, Aisen P, Greenberg S (2023). Lecanemab: Appropriate Use Recommendations. J Prev Alzheimers Dis.

[3] Budd Haeberlein S, Aisen PS, Barkhof F, Chalkias S, Chen T, Cohen S (2022). Two Randomized Phase 3 Studies of Aducanumab in Early Alzheimer's Disease. J Prev Alzheimers Dis.

[4] van Dyck CH, Swanson CJ, Aisen P, Bateman RJ, Chen C, Gee M (2023). Lecanemab in Early Alzheimer's Disease. N Engl J Med.

[5] Sims JR, Zimmer JA, Evans CD, Lu M, Ardayfio P, Sparks J, et al. Donanemab in Early Symptomatic Alzheimer Disease: The Trailblazer-alz 2 randomized clinical trial. JAMA. 2023;330(6):512–27. 10.1001/jama.2023.13239 PMC10352931 37459141

[6] Sims JR, Iwatsubo T, Greenberg SM, Mintun M, Atri A, Zimmer JA, et al. S2- Donanemab In early symptomatic alzheimer’s disease: additional insights from TRAILBLAZER-ALZ 2. 16th Clinical Trials on alzheimer’s disease (CTAD) Boston, MA (USA) October 24–27, 2023: Symposia. J Prev Alzheimers Dis. 2023;10(1):S6–7.

[7] Van Dyck CH, Johnson K, Sperling R, Irizarry M. S4- Lecanemab for early alzheimer’ s disease:long-term outcomes, predictive biomarkers and novel subcutaneous administration. 16th Clinical trials on alzheimer’s disease (CTAD) Boston, MA (USA) October 24–27, 2023: Symposia. J Prev Alzheimers Dis. 2023;10(1):S9–11.

[8] Parnetti L, Chipi E, Salvadori N, D'Andrea K, Eusebi P (2019). Prevalence and risk of progression of preclinical Alzheimer's disease stages: a systematic review and meta-analysis. Alzheimers Res Ther.

[9] Leuzy A, Mattsson-Carlgren N, Palmqvist S, Janelidze S, Dage JL, Hansson O (2022). Blood-based biomarkers for Alzheimer's disease. EMBO Mol Med.

[10] Teunissen CE, Verberk IMW, Thijssen EH, Vermunt L, Hansson O, Zetterberg H (2022). Blood-based biomarkers for Alzheimer's disease: towards clinical implementation. Lancet Neurol.

[11] Nakamura A, Kaneko N, Villemagne VL, Kato T, Doecke J, Doré V (2018). High performance plasma amyloid-β biomarkers for Alzheimer's disease. Nature.

[12] Schindler SE, Bollinger JG, Ovod V, Mawuenyega KG, Li Y, Gordon BA (2019). High-precision plasma β-amyloid 42/40 predicts current and future brain amyloidosis. Neurology.

[13] Pannee J, Törnqvist U, Westerlund A, Ingelsson M, Lannfelt L, Brinkmalm G (2014). The amyloid-β degradation pattern in plasma—A possible tool for clinical trials in Alzheimer's disease. Neurosci Lett.

[14] Gonzalez-Ortiz F, Kac PR, Brum WS, Zetterberg H, Blennow K, Karikari TK (2023). Plasma phospho-tau in Alzheimer’s disease: towards diagnostic and therapeutic trial applications. Mol Neurodegener.

[15] Schindler SE, Karikari TK, Ashton NJ, Henson RL, Yarasheski KE, West T (2022). Effect of Race on Prediction of Brain Amyloidosis by Plasma Aβ42/Aβ40, Phosphorylated Tau, and Neurofilament Light. Neurology.

[16] Hajjar I, Yang Z, Okafor M, Liu C, Waligorska T, Goldstein FC (2022). Association of Plasma and Cerebrospinal Fluid Alzheimer Disease Biomarkers With Race and the Role of Genetic Ancestry, Vascular Comorbidities, and Neighborhood Factors. JAMA Netw Open.

[17] Saji N, Sakurai T, Suzuki K, Mizusawa H, Toba K (2016). ORANGE's challenge: developing wide-ranging dementia research in Japan. Lancet Neurol.

[18] Sato K, Ihara R, Suzuki K, Niimi Y, Toda T, Jimenez-Maggiora G (2021). Predicting amyloid risk by machine learning algorithms based on the A4 screen data: Application to the Japanese Trial-Ready Cohort study. Alzheimers Dement (N Y).

[19] Walsh SP, Raman R, Jones KB, Aisen PS (2006). ADCS Prevention Instrument Project: the Mail-In Cognitive Function Screening Instrument (MCFSI). Alzheimer Dis Assoc Disord.

[20] Donohue MC, Sperling RA, Salmon DP, Rentz DM, Raman R, Thomas RG (2014). The preclinical Alzheimer cognitive composite: measuring amyloid-related decline. JAMA Neurol.

[21] Palmqvist S, Janelidze S, Quiroz YT, Zetterberg H, Lopera F, Stomrud E (2020). Discriminative Accuracy of Plasma Phospho-tau217 for Alzheimer Disease vs Other Neurodegenerative Disorders. JAMA.

[22] Janelidze S, Teunissen CE, Zetterberg H, Allué JA, Sarasa L, Eichenlaub U (2021). Head-to-Head Comparison of 8 Plasma Amyloid-β 42/40 Assays in Alzheimer Disease. JAMA Neurol.

[23] Janelidze S, Berron D, Smith R, Strandberg O, Proctor NK, Dage JL (2021). Associations of Plasma Phospho-Tau217 Levels With Tau Positron Emission Tomography in Early Alzheimer Disease. JAMA Neurol.

[24] Janelidze S, Palmqvist S, Leuzy A, Stomrud E, Verberk IMW, Zetterberg H (2022). Detecting amyloid positivity in early Alzheimer's disease using combinations of plasma Aβ42/Aβ40 and p-tau. Alzheimers Dement.

[25] Ikari Y, Akamatsu G, Nishio T, Ishii K, Ito K, Iwatsubo T (2016). Phantom criteria for qualification of brain FDG and amyloid PET across different cameras. EJNMMI Phys.

[26] Klunk WE, Koeppe RA, Price JC, Benzinger TL, Devous MD, Jagust WJ (2015). The Centiloid Project: Standardizing quantitative amyloid plaque estimation by PET. Alzheimers Dement.

[27] Hanon O, Vidal JS, Lehmann S, Bombois S, Allinquant B, Baret-Rose C (2022). Plasma amyloid beta predicts conversion to dementia in subjects with mild cognitive impairment: The BALTAZAR study. Alzheimers Dement.

[28] Angioni D, Delrieu J, Hansson O, Fillit H, Aisen P, Cummings J (2022). Blood Biomarkers from Research Use to Clinical Practice: What Must Be Done? A Report from the EU/US CTAD Task Force. J Prev Alzheimers Dis.

[29] Thijssen EH, La Joie R, Strom A, Fonseca C, Iaccarino L, Wolf A (2021). Plasma phosphorylated tau 217 and phosphorylated tau 181 as biomarkers in Alzheimer's disease and frontotemporal lobar degeneration: a retrospective diagnostic performance study. Lancet Neurol.

[30] Janelidze S, Bali D, Ashton NJ, Barthélemy NR, Vanbrabant J, Stoops E (2023). Head-to-head comparison of 10 plasma phospho-tau assays in prodromal Alzheimer's disease. Brain.

[31] Jonaitis EM, Janelidze S, Cody KA, Langhough R, Du L, Chin NA (2023). Plasma phosphorylated tau 217 in preclinical Alzheimer's disease. Brain Commun..

[32] Milà-Alomà M, Ashton NJ, Shekari M, Salvadó G, Ortiz-Romero P, Montoliu-Gaya L (2022). Plasma p-tau231 and p-tau217 as state markers of amyloid-β pathology in preclinical Alzheimer's disease. Nat Med.

[33] Ashton NJ, Janelidze S, Mattsson-Carlgren N, Binette AP, Strandberg O, Brum WS (2022). Differential roles of Aβ42/40, p-tau231 and p-tau217 for Alzheimer's trial selection and disease monitoring. Nat Med.

[34] Salvadó G, Ossenkoppele R, Ashton NJ, Beach TG, Serrano GE, Reiman EM (2023). Specific associations between plasma biomarkers and postmortem amyloid plaque and tau tangle loads. EMBO Mol Med.

[35] Rissman RA, Langford O, Raman R, Donohue MC, Abdel-Latif S, Meyer MR, et al. Plasma Aβ42/Aβ40 and phospho-tau217 concentration ratios increase the accuracy of amyloid PET classification in preclinical Alzheimer's disease. Alzheimers Dement. 2024;20(2):1214–24. 10.1002/alz.13542 PMC10916957 37932961

[36] Palmqvist S, Stomrud E, Cullen N, Janelidze S, Manuilova E, Jethwa A (2023). An accurate fully automated panel of plasma biomarkers for Alzheimer's disease. Alzheimers Dement.

[37] Miyashita A, Kikuchi M, Hara N, Ikeuchi T (2023). Genetics of Alzheimer's disease: an East Asian perspective. J Hum Genet.

[38] Raman R, Quiroz YT, Langford O, Choi J, Ritchie M, Baumgartner M (2021). Disparities by Race and Ethnicity Among Adults Recruited for a Preclinical Alzheimer Disease Trial. JAMA Netw Open.

