# Supplementary material for: Combining plasma Aβ and p-tau217 improves detection of brain amyloid in non-demented elderly
Source: Alzheimers Res Ther. 2024 May 23;16:115. doi: 10.1186/s13195-024-01469-w (PMC11112892; doi:10.1186/s13195-024-01469-w)
Supplement: Supplementary file 1 — Supplementary Material 1. Supplemental Figure 1 Nomograms for the logistic regression analysis to detect Aβ-PET positivity in the J-TRC cohort. CI: clinical information including age, sex, and APOE, CDR: clinical dementia rating (global score). Supplemental Figure 2 Nomograms for the logistic regression analysis to detect Aβ-PET positivity in the BioFINDER cohort. CI: clinical information including age, sex, and APOE, CU: cognitively unimpaired, MCI: mild cognitive impairment. [file 13195_2024_1469_MOESM1_ESM.pptx]

## Slide 1
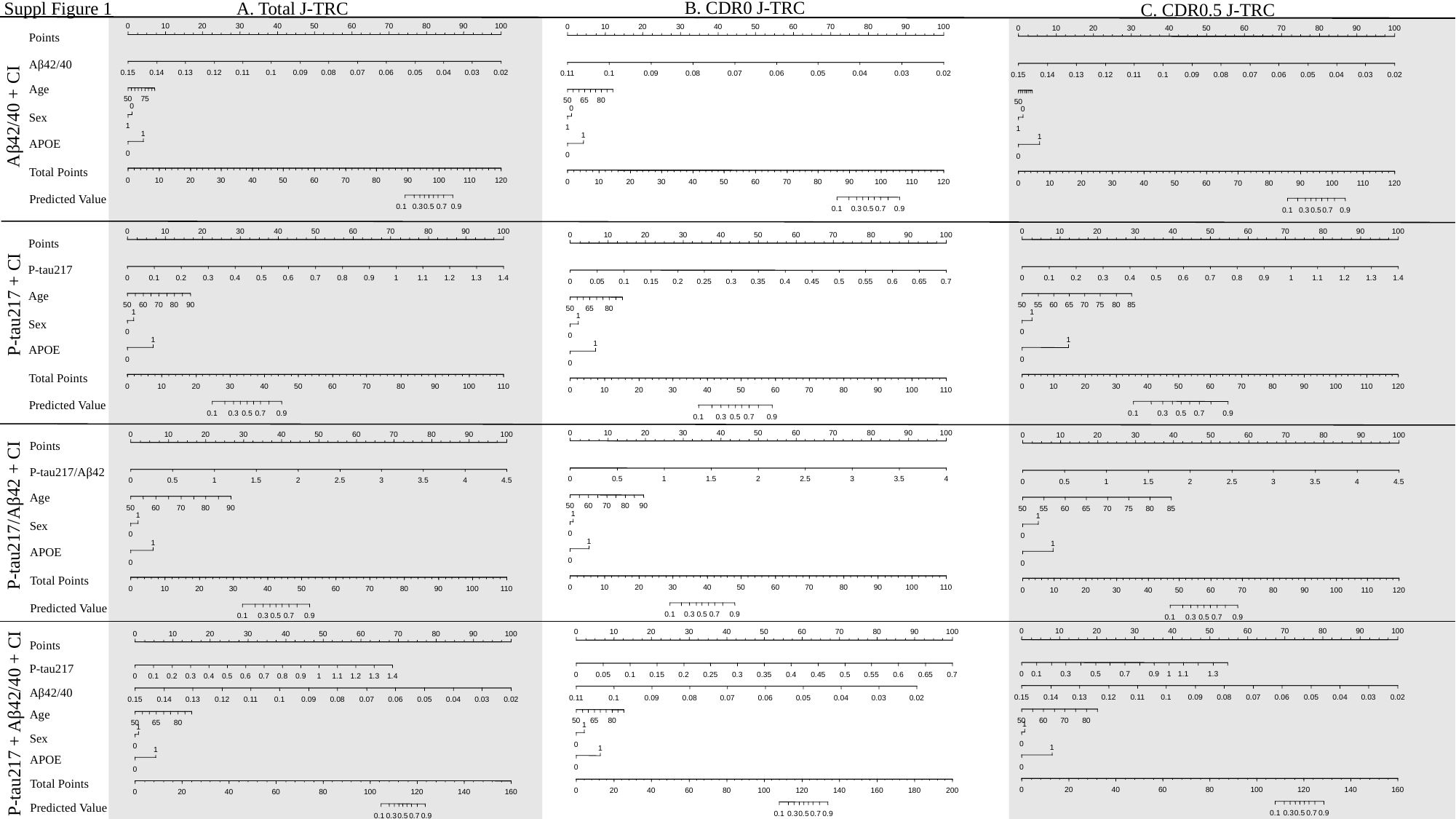

Suppl Figure 1
B. CDR0 J-TRC
A. Total J-TRC
C. CDR0.5 J-TRC
0
10
20
30
40
50
60
70
80
90
100
0.11
0.1
0.09
0.08
0.07
0.06
0.05
0.04
0.03
0.02
50
65
80
0
1
1
0
0
10
20
30
40
50
60
70
80
90
100
110
120
0.1
0.3
0.5
0.7
0.9
0
10
20
30
40
50
60
70
80
90
100
Points
Aβ42/40
0.15
0.14
0.13
0.12
0.11
0.1
0.09
0.08
0.07
0.06
0.05
0.04
0.03
0.02
Age
50
75
0
Sex
1
1
APOE
0
Total Points
0
10
20
30
40
50
60
70
80
90
100
110
120
Predicted Value
0.1
0.3
0.5
0.7
0.9
0
10
20
30
40
50
60
70
80
90
100
0.15
0.14
0.13
0.12
0.11
0.1
0.09
0.08
0.07
0.06
0.05
0.04
0.03
0.02
50
0
1
1
0
0
10
20
30
40
50
60
70
80
90
100
110
120
0.1
0.3
0.5
0.7
0.9
Aβ42/40 + CI
0
10
20
30
40
50
60
70
80
90
100
Points
P-tau217
0
0.1
0.2
0.3
0.4
0.5
0.6
0.7
0.8
0.9
1
1.1
1.2
1.3
1.4
Age
50
60
70
80
90
1
Sex
0
1
APOE
0
Total Points
0
10
20
30
40
50
60
70
80
90
100
110
Predicted Value
0.1
0.3
0.5
0.7
0.9
0
10
20
30
40
50
60
70
80
90
100
0
0.05
0.1
0.15
0.2
0.25
0.3
0.35
0.4
0.45
0.5
0.55
0.6
0.65
0.7
50
65
80
1
0
1
0
0
10
20
30
40
50
60
70
80
90
100
110
0.1
0.3
0.5
0.7
0.9
0
10
20
30
40
50
60
70
80
90
100
0
0.1
0.2
0.3
0.4
0.5
0.6
0.7
0.8
0.9
1
1.1
1.2
1.3
1.4
50
55
60
65
70
75
80
85
1
0
1
0
0
10
20
30
40
50
60
70
80
90
100
110
120
0.1
0.3
0.5
0.7
0.9
P-tau217 + CI
0
10
20
30
40
50
60
70
80
90
100
Points
P-tau217/Aβ42
0
0.5
1
1.5
2
2.5
3
3.5
4
4.5
Age
50
60
70
80
90
1
Sex
0
1
APOE
0
Total Points
0
10
20
30
40
50
60
70
80
90
100
110
Predicted Value
0.1
0.3
0.5
0.7
0.9
0
10
20
30
40
50
60
70
80
90
100
0
0.5
1
1.5
2
2.5
3
3.5
4
4.5
50
55
60
65
70
75
80
85
1
0
1
0
0
10
20
30
40
50
60
70
80
90
100
110
120
0.1
0.3
0.5
0.7
0.9
0
10
20
30
40
50
60
70
80
90
100
0
0.5
1
1.5
2
2.5
3
3.5
4
50
60
70
80
90
1
0
1
0
0
10
20
30
40
50
60
70
80
90
100
110
0.1
0.3
0.5
0.7
0.9
P-tau217/Aβ42 + CI
0
10
20
30
40
50
60
70
80
90
100
0
0.05
0.1
0.15
0.2
0.25
0.3
0.35
0.4
0.45
0.5
0.55
0.6
0.65
0.7
0.11
0.1
0.09
0.08
0.07
0.06
0.05
0.04
0.03
0.02
50
65
80
1
0
1
0
0
20
40
60
80
100
120
140
160
180
200
0.1
0.3
0.5
0.7
0.9
0
10
20
30
40
50
60
70
80
90
100
0
0.1
0.3
0.5
0.7
0.9
1
1.1
1.3
0.15
0.14
0.13
0.12
0.11
0.1
0.09
0.08
0.07
0.06
0.05
0.04
0.03
0.02
50
60
70
80
1
0
0
1
0
20
40
60
80
100
120
140
160
0.1
0.3
0.5
0.7
0.9
0
10
20
30
40
50
60
70
80
90
100
Points
P-tau217
0
0.1
0.2
0.3
0.4
0.5
0.6
0.7
0.8
0.9
1
1.1
1.2
1.3
1.4
Aβ42/40
0.15
0.14
0.13
0.12
0.11
0.1
0.09
0.08
0.07
0.06
0.05
0.04
0.03
0.02
Age
50
65
80
1
Sex
0
APOE
1
0
Total Points
0
20
40
60
80
100
120
140
160
Predicted Value
0.1
0.3
0.5
0.7
0.9
P-tau217 + Aβ42/40 + CI

## Slide 2
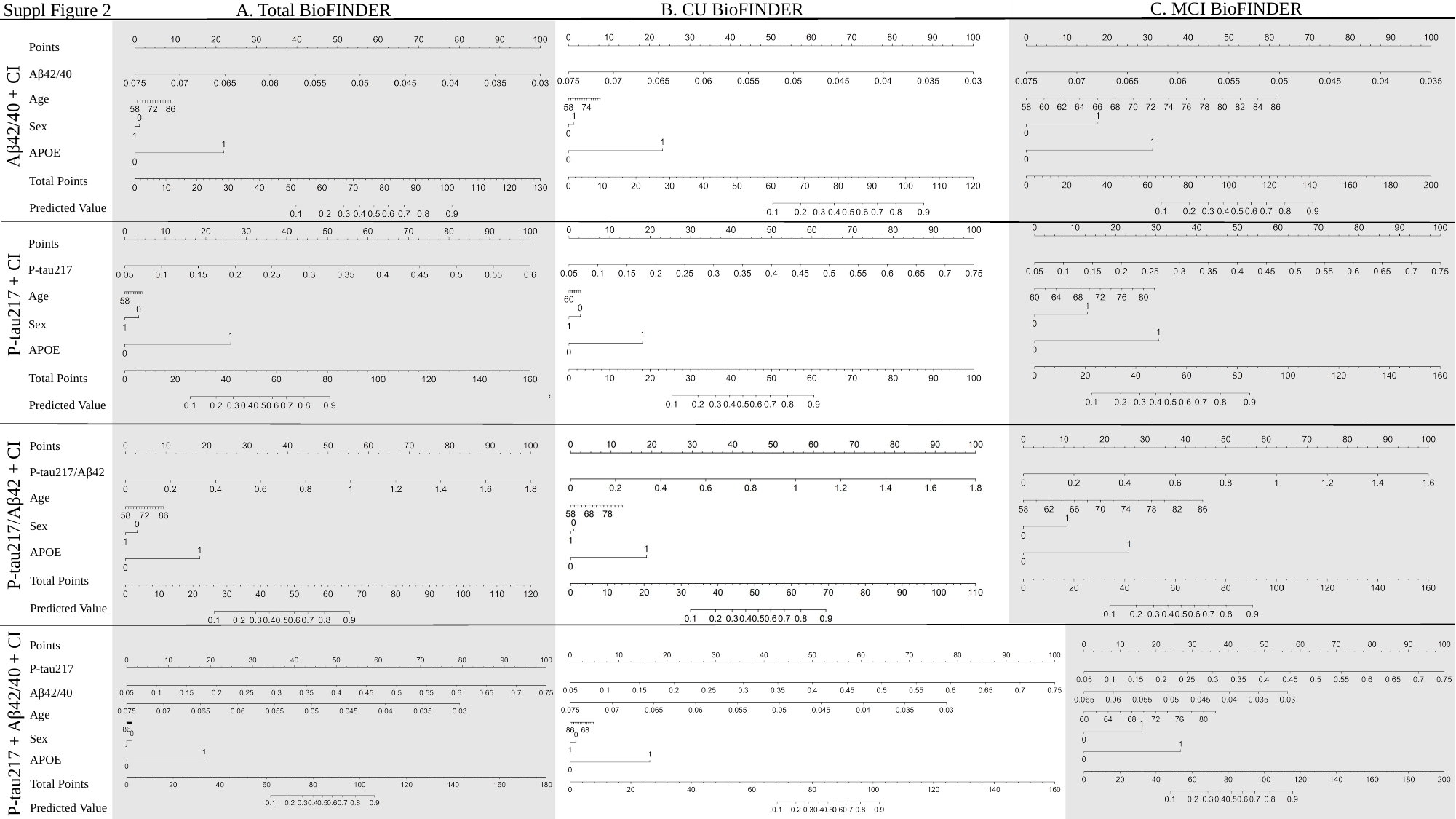

Suppl Figure 2
C. MCI BioFINDER
B. CU BioFINDER
A. Total BioFINDER
Points
Aβ42/40
Age
Aβ42/40 + CI
Sex
APOE
Total Points
Predicted Value
Points
P-tau217
Age
P-tau217 + CI
Sex
APOE
Total Points
Predicted Value
Points
P-tau217/Aβ42
Age
P-tau217/Aβ42 + CI
Sex
APOE
Total Points
Predicted Value
Points
P-tau217
Aβ42/40
Age
P-tau217 + Aβ42/40 + CI
Sex
APOE
Total Points
Predicted Value
